# The Disinfection of Scarlet Fever and Other Infectious Diseases by Antiseptic Inunction

**Published:** 1892-12

**Authors:** 


*The Disinfection of Scarlet Fever and other Infectious Diseases by Antiseptic Inunction.* By J. BRENDON CURGENVEN, M.R.C.S., L.S.A. Pp. 25. London: H. K. LEWIS. 1891.

In this essay the author claims that scarlet fever, if treated by inunction with the essential oil of eucalyptus, may be prevented from spreading without isolation, and, as a rule, in ten days. This method of treatment was, we believe, first discussed at a meeting of the Epidemiological Society in 1890, and the present essay was read at the Congress of Hygiene in 1891.

We have re-read the essay with considerable interest, but must confess that the author does not yet appear to have proved his case, as the number of successful instances quoted in support is too small for accurate conclusions. The question is, however, worthy of more than passing notice, and there would seem to be no objection to the adoption of this method of inunction as a preventive treatment where isolation is difficult to obtain.

